# The peripheral vascular responses in non‐freezing cold injury and matched controls

**DOI:** 10.1113/EP090721

**Published:** 2023-02-19

**Authors:** Clare M. Eglin, Jennifer Wright, Matthew J. Maley, Sarah Hollis, Heather Massey, Hugh Montgomery, Michael J. Tipton

**Affiliations:** ^1^ Extreme Environments Laboratory, School of Sport, Health and Exercise Science University of Portsmouth Portsmouth UK; ^2^ Environmental Ergonomics Research Centre, Loughborough School of Design and Creative Arts Loughborough University Loughborough UK; ^3^ Regional Occupational Health Team (ROHT) Catterick Catterick Garrison UK; ^4^ Department of Medicine University College London London UK

**Keywords:** cold injury, pathophysiology, vascular

## Abstract

The impact of non‐freezing cold injury (NFCI) on peripheral vascular function was investigated. Individuals with NFCI (NFCI group) and closely matched controls with either similar (COLD group) or limited (CON group) previous cold exposure were compared (*n* = 16). Peripheral cutaneous vascular responses to deep inspiration (DI), occlusion (PORH), local cutaneous heating (LH) and iontophoresis of acetylcholine and sodium nitroprusside were investigated. The responses to a cold sensitivity test (CST) involving immersion of a foot in 15°C water for 2 min followed by spontaneous rewarming, and a foot cooling protocol (footplate cooled from 34°C to 15°C), were also examined. The vasoconstrictor response to DI was lower in NFCI compared to CON (toe: 73 (28)% vs. 91 (17)%; *P* = 0.003). The responses to PORH, LH and iontophoresis were not reduced compared to either COLD or CON. During the CST, toe skin temperature rewarmed more slowly in NFCI than COLD or CON (10 min: 27.4 (2.3)°C vs. 30.7 (3.7)°C and 31.7 (3.9)°C, *P* < 0.05, respectively); however, no differences were observed during the footplate cooling. NFCI were more cold‐intolerant (*P* < 0.0001) and reported colder and more uncomfortable feet during the CST and footplate cooling than COLD and CON (*P* < 0.05). NFCI showed a decreased sensitivity to sympathetic vasoconstrictor activation than CON and greater cold sensitivity (CST) compared to COLD and CON. None of the other vascular function tests indicated endothelial dysfunction. However, NFCI perceived their extremities to be colder and more uncomfortable/painful than the controls.

## Abstract (highlights)

- **What is the central question of this study?**
  Does non‐freezing cold injury (NFCI) alter normal peripheral vascular function?
- **What is the main finding and its importance?**
  Individuals with NFCI were more cold sensitive (rewarmed more slowly and felt more discomfort) than controls. Vascular tests indicated that extremity endothelial function was preserved with NFCI and that sympathetic vasoconstrictor response might be reduced. The pathophysiology underpinning the cold sensitivity associated with NFCI thus remains to be identified.

## 1 INTRODUCTION

Non‐freezing cold injury (NFCI) is caused by prolonged exposure to cold (and often cold/wet) environments and predominantly affects the hands and feet (Golden et al., 2013; Kuht et al., 2018; Ungley et al., 1945). NFCI is more prevalent in the context of military service, where it is a common form of non‐combat‐related injury in cold and temperate environments during both conflicts and peacetime (Golden et al., 2013; Ministry of Defence, 2018; Oakley, 2009). Although less reported in civilian populations, exposure to cold in occupations such as farming, fishing, construction, cold storage and food preparation may lead to NFCI. In addition, the increased popularity of outdoor recreational activities also increases the risk of cold injuries and NFCI has been reported in paddle sports (Oakley et al., 2022), ice skaters (Tlougan et al., 2011), cyclists (Fraser et al., 1979), divers (Laden et al., 2007), long distance Polar rowers (Longman et al., 2020), and hikers who become incapacitated (Paal et al., 2013).

During World War II, Ungley et al. (1945) identified four stages of NFCI in shipwrecked mariners. In stage 1, during cold exposure, the tissue is ischaemic and numb. During rewarming (stage 2) the tissue becomes mottled blue and painful. In stage 3, the tissue becomes swollen, red and hot and the associated pain may be persistent and severe; this hyperaemic stage may last for up to 4 weeks in severe cases. Stage 4 is the chronic state, which may last for many years and is characterised (in variable combination and severity) by cold sensitivity, numbness, hyperhidrosis and persistent, intractable pain (Golden et al., 2013; Ungley et al., 1945). Other neurological symptoms may also occur, including paraesthesia, loss of proprioception and, in severe cases, impaired neuromuscular function.

The current phenotype of NFCI is typically less severe than that described during the first and second world wars (Kuht et al., 2018; Ungley et al., 1945). The long‐term symptoms of NFCI most commonly seen are persistent pain, impaired sensory perception and cold sensitivity (Eglin et al., 2013; Vale et al., 2017). Cold sensitivity may be associated with protracted peripheral vasoconstriction leading to an increase in peripheral cooling and resultant pain and numbness, sequelae which may increase the risk of further cold injury (Golden et al., 2013; Jørum & Opstad, 2019). These chronic symptoms of NFCI can have a profound effect on an individual's quality of life and employability.

There is a paucity of research into NFCI, and its pathogenesis and pathophysiology are thus poorly understood. The lack of information on NFCI means that there is no valid diagnostic test. As a result, the prevention, assessment and treatment of NFCI is poorly evidence‐based. Animal and human studies suggest that NFCI involves damage to nerve fibres (Anand et al., 2017; Jørum & Opstad, 2019; Vale et al., 2017) and the microvasculature (Eglin et al., 2013; Stephens et al., 2009), although these have generally been investigated separately and without appropriate controls. This is important as there is probably a broad range in the ‘normal’ response to peripheral cooling (Haman et al., 2022) with a large proportion of the general population being cold sensitive as a result of previous cold exposure (Ahle et al., 1990; Eglin et al., 2017; Eglin et al., 2020; Hope et al., 2014). In addition, individuals from an African/Caribbean background are more susceptible to NFCI (Kuht et al., 2018) and it is likely that women are at greater risk than men (Donnell & Taubman, 2011) due to their greater rate of hand and foot cooling in the cold (Lunt & Tipton, 2014).

This is the first study to investigate the physiological features of NFCI, with individuals suffering NFCI (NFCI group) being compared to controls matched for race, age, sex and physical characteristics. Two control groups were employed, one with similar previous cold exposure (COLD) to the NFCI group, and one with minimal cold exposure (CON). Subsequent papers will describe the associated changes in neural responses (Wright et al., 2023), and biomarkers of endothelial function and damage, redox signalling and inflammation status (Eglin et al., 2023). In this, the first report from the study, we describe the changes in peripheral vascular response associated with NFCI. It was hypothesised that the NFCI group would have impaired peripheral vascular function when compared to both COLD and CON and that COLD would have a reduced peripheral vascular function when compared to CON.

## 2 METHODS

The protocol received ethical approval from the Ministry of Defence Research Ethics Committee (909/MoDREC/18), and all participants gave informed written consent prior to any testing. The study complied with the *Declaration of Helsinki* (1964), as last revised at the 64th World Medical Association General Assembly, Brazil, 2013, except for registration in a database.

Testing was undertaken between January 2019 and October 2019 when the ambient outdoor temperature was 9.5 (5.1)°C. Given the need to recruit the NFCI group first to enable matching of the Control participants, the CON group were tested later when the ambient temperature was higher (NFCI 5.9°C (range −3 to 12°C); COLD 8.2°C (range 3–18°C); CON 12.8°C (range 5–22°C); *P* < 0.001). An a priori sample size calculation was performed using G*Power based on data from previously published results of vascular tests (peak cutaneous vascular conductance during post‐occlusive reactive hyperaemia (Stoyneva, 2016) and iontophoresis of acetylcholine (Maley et al., 2017) and toe skin temperature during the cold sensitivity test (Eglin, 2011)). For 80% power and an α‐level of 5%, it was calculated that between eight and 17 participants were required in each group.

The NFCI group were recruited from a regional UK military cold injuries clinic and did not have a history of frostbite. NFCI diagnosis was based on a detailed history of the estimated degree of cold exposure, symptoms at point of injury, persistence of symptoms and standard primary care level neurological examination, and bench‐marked against a standardised set of diagnostic criteria (defined from a mix of animal and human research evidence and case series, see Ministry of Defence, 2021). Those with Raynaud's phenomenon were excluded using the International Consensus Criteria for the Diagnosis of Raynaud's Phenomenon. Assessments were conducted by a medical doctor (S.H.) with over 20 years of experience in reviewing NFCI cases and 7 years of running the Defence Medical Services Regional NFCI Clinic.

Cold‐exposed controls (COLD) with no previous diagnosis of NFCI were recruited from UK Army soldiers. Two COLD participants had a previous NFCI but were now considered, by the same medical doctor (S.H.), to be completely recovered. The volunteers were selected to match the NFCI group for cold exposure, sex, race, age, aerobic fitness and body mass index (BMI) as closely as possible.

Controls (CON) with limited previous exposure to cold and no previous diagnosis of NFCI were recruited from students and staff at the University of Portsmouth. The volunteers were selected to match the NFCI group for sex, race, age, aerobic fitness and BMI as closely as possible. It was established through a verbal screening process during recruitment that CON participants did not partake in any sports/activities where they were likely to get cold (i.e., they partake in indoor sport/gym activities), and that they had not encountered any notable events of being cold where they may have sustained a cold injury.

Participants attended the laboratory wearing T‐shirt and trousers on three occasions (visits A, B and C). In visit A, anthropometric measurements were recorded and a submaximal fitness test performed. In visit B, the participant was seated in a quiet room at 24°C whilst the post‐occlusive reactive hyperaemic response was studied, as were the microvascular responses to deep inspiration, cutaneous local heating and acetylcholine and sodium nitroprusside iontophoresis. Throughout all the testing, participants were asked to remain as still as possible. Skin temperature was measured on the dorsal aspect of the hand, volar aspect of the forearm, calf and dorsum of the foot using skin thermistors (EUS‐U, Grant Instruments, Cambridge, UK) and recorded using a data logger (Squirrel 2020, Grant Instruments). In visit C, participants performed a deep inspiration protocol and a cold sensitivity test in 30°C air, followed by a foot cooling protocol in 25°C air. For logistical reasons, the time of day that the participants undertook each session was not controlled (however, there was no between‐group difference in the time of day at which the participants undertook their tests) and some participants undertook more than one session in a day with a total of 17 participants completing all of their testing on a single day. If conducted on the same day, visit A and B were always conducted before visit C, since this involved tests that altered skin temperature which might have impacted on the responses to the other tests. The order of the individual tests within each visit was the same for all participants.

Prior to arrival at the laboratory, participants were asked to refrain from smoking, or ingestion of caffeine or over‐the‐counter painkillers such as paracetamol, aspirin and ibuprofen for 8 h. Participants were instructed not to undertake heavy exercise and to refrain from alcohol consumption for 24 h. Participants were also requested to avoid eating food high in nitrate for the 2 days before testing.

In visit A, participants’ height (SECA 213, SECA, Birmingham, UK), mass (SECA 899) and foot volume using the water displacement method were measured. Participants then undertook an Åstrand‐Rhyming submaximal cycle test on a cycle ergometer (Lode Corival CPET, Gronigen, The Netherlands) to estimate their aerobic fitness (ACSM, 2010). A venous blood sample was taken for subsequent analysis at a local hospital for blood cell count and serum concentrations of cholesterol, triglycerides and HbA1c.

### 2.1 Deep inspiration

After a 15 min period of acclimatisation to the environment (ambient temperature 24.1 (0.8)°C, relative humidity 46 (9)%), participants were asked to take a rapid, deep breath to maximum inspiratory capacity and hold it for 10 s followed by normal breathing. A practice run was conducted, after which three repeated breath‐holds were undertaken at 3 min intervals (Mueck‐Weymann & Rauh, 2002). Skin blood flow of the left thumb and great toe pad were measured before and following the respiratory manoeuvres using a single channel laser Doppler probe (VP12, Moor Instruments, Axminster, UK) inserted into an integrated heater (VHP2, Moor Instruments) clamping local skin temperature at 33°C. Outputs from the laser Doppler meter (MoorVMS‐LDF2, Moor Instruments) and heater were recorded and analysed using dedicated software (MoorVMS‐PC, v4.2, Moor Instruments). Blood pressure was recorded before and after the three respiratory manoeuvres using an automated blood pressure meter (M6 AC, Omron, Kyoto, Japan) on the contralateral arm to enable calculation of mean arterial pressure (MAP) and cutaneous vascular conductance (CVC: flux/MAP). Inspiratory gasp vascular response (IGVR) was calculated as the percentage decrease in CVC from baseline (Mayrovitz & Groseclose, 2002) with the maximum of the three IGVRs being reported.

### 2.2 Post‐occlusive reactive hyperaemia

Post‐occlusive reactive hyperaemia (PORH) was assessed using an established protocol (Roustit et al., 2010; Barwick et al., 2015) in an ambient temperature and relative humidity of 24.3 (0.6)°C and 45 (10)%, respectively. Following the deep inspiration protocol, the laser Doppler probes were kept in the same location with skin temperature clamped at 33°C for 5 min using the integrated heater. Blood flow to the great toe and thumb was then occluded for 3 min using 2.5 cm pneumatic cuffs (placed proximal to the laser Doppler probe, around the base of the digit) rapidly inflated to 220 mmHg using a pressure cuff controller (Moor‐VMS‐Pres, Moor Instruments). The cuffs were then rapidly released with the PORH response measured for a further 5 min. Blood pressure was measured from the contralateral brachial artery before and after occlusion. The peak response was reported as a percentage of baseline (flux_peak_/flux_baseline_ × 100) where flux_baseline_ was taken as the mean flux over 60 s of stable recording prior to occlusion. PORH index was calculated by dividing AUC_peak_ by AUC_baseline_ (area under the curve (AUC)) during the 60 s immediately after the end of occlusion and during the 60 s immediately prior to start of occlusion, respectively). The area of hyperaemia (AUC following release of occlusion) was calculated using VMS software (Moor Instruments).

### 2.3 Cutaneous local heating

The responses to cutaneous local heating were investigated using the protocol described previously (Minson et al., 2002; Fieger & Wong, 2010). Following the PORH protocol, in a room controlled at 24.3 (0.6)°C and 45 (10)% relative humidity, the laser Doppler probes were kept in the same location with skin temperature clamped at 33°C for 10 min. Local temperature was then increased by 0.1°C/s to 42°C and maintained at this temperature for 20 min. Skin temperature was then increased by 0.1°C/s to 44°C and maintained at this temperature for 15 min. Blood pressure was measured from the contralateral brachial artery every 10 min. The initial peak and nadir flux were calculated from the highest and lowest values, respectively, over 30 s periods. Plateau averages at 42°C and 44°C were taken from stable 5 min periods and these values were expressed as a percentage of baseline CVC taken over a stable 5 min period. Ratio of the AUC at baseline (measured over 5 min but corrected to 20 min) and the AUC during the 20 min at 42°C was also calculated (AUC ratio).

### 2.4 Iontophoresis

Following the cutaneous heating protocol, acetylcholine (ACh 1% w/v) and sodium nitroprusside (SNP 0.1% w/v) were delivered transdermally using iontophoresis to the volar aspect of the forearm (to determine any systemic changes in endothelial function) at 24.3 (0.8)°C and 45 (9)% relative humidity, followed by the dorsal aspect of the foot at 24.4 (0.7)°C and 47 (12)%. Following gentle cleaning of the skin with sterile water, two Perspex chambers (8 mm inner diameter) containing either the anode or the cathode were attached to the skin sites avoiding any obvious large blood vessels or tattoos. Both electrodes were connected to a battery‐powered iontophoresis controller (MIC 2, Moor Instruments). The anode chamber was placed distally on the forearm and then laterally on the dorsum of the foot and filled with approximately 0.5 ml of ACh. The cathode chamber was placed proximally on the forearm and then medially on the dorsum of the foot and filled with approximately 0.5 ml of SNP. Both ACh (Sigma‐Aldrich, St Louis, MO, USA) and SNP (Sigma‐Aldrich) were diluted with sterile water and stock solutions were kept refrigerated in darkened bottles with fresh solutions made every 10 days. The iontophoresis protocol was the same as that reported previously (Eglin et al., 2017), and consisted of four pulses of 25 μA followed by one pulse of 50 μA, one of 100 μA, one of 150 μA and a final pulse of 200 μA. All pulses were applied for 20 s with 120‐s intervals between each pulse where no current was applied. Average flux over the 60 s period following each pulse was recorded and subsequently converted to CVC. After an interval of 5 min, the protocol was repeated on the foot. Blood flow was measured using a laser Doppler probe (VP12, Moor Instruments) inserted into the iontophoresis chamber and recorded using VMS software. Skin temperature of the iontophoretic site was maintained at 33°C using an integrated heater (VHP2, Moor Instruments) and the forearm and foot were placed on a heater pad (HK35, Beurer, Ulm, Germany) to maintain a warm skin temperature. Blood pressure was measured from the contralateral brachial artery prior to and following acquisition of each ACh and SNP dose–response curve.

Maximum CVC and AUC following transdermal delivery of ACh and SNP (with a cumulative current of 600 μA) on the forearm were analysed. As reported previously (Maley et al., 2015), some participants had a high skin resistance and therefore could not receive the full current during iontophoresis on the foot. Therefore, maximum CVC and AUC responses to ACh and SNP on the foot for pulses up to a cumulative current of 100 μA were analysed.

### 2.5 Cold sensitivity test

The cold sensitivity test (CST) has been described previously (Eglin et al., 2013; Hope et al., 2014; Maley et al., 2014; Eglin et al., 2017). Participants entered a climatic chamber controlled at an air temperature of 30.4 (0.9)°C and relative humidity of 38 (6)%, removed their shoes and socks and rested in a semi‐recumbent position for 5 min. At the end of the 5 min period, skin temperatures of the toe pads were measured using a thermal imaging camera (A655sc, FLIR Systems, Wilsonville, OR, USA) positioned at a distance of 1 m and perpendicular to the soles of the feet according to the guidelines described by Moreira et al. (2017). Participants then exercised on a cycle ergometer (Lode Corival CPET) for 12 min at an external work rate of 50 W.

Participants then undertook the deep inspiration protocol as described above, except that they remained at an ambient temperature of 30.3 (2.0)°C and relative humidity of 40 (11)%. Following this, participants rested in a seated position for at least 5 min for measurement of baseline data. Participants then placed their left foot in a plastic bag and immersed it into stirred water at 15.0 (0.1)°C to the point of their mid‐malleoli for 2 min. After the immersion period, the plastic bag was removed and spontaneous rewarming was monitored for 10 min whilst the participant remained resting in a semi‐recumbent position. Ambient temperature and relative humidity were controlled at 30.4 (1.8)°C and 41 (7)%, respectively, during the CST with air temperature adjacent (within 10 cm) to the foot averaging 29.9 (1.1)°C. Skin blood flow of both great toe pads was measured throughout the test using a multi‐channel laser Doppler probe (VP1T/7, Moor Instruments) and skin temperature of the toe pads was recorded before immersion and throughout the rewarming using the infrared camera. Mean skin temperature of all the toe pads, the skin temperature of the great toe and the skin temperature of the coldest toe pad before foot immersion and 5 and 10 min after immersion (rewarming) were used for analysis. Thermal comfort and sensation of the left foot were recorded before immersion, during immersion and after 5 and 10 min of rewarming using a 20 cm visual analogue scale where 0 = very uncomfortable/cold and 20 = very comfortable/hot (Zhang et al., 2004). Pain sensation in the left foot was measured at the same time points using a 0–10 numerical rating scale where: 0 = no pain, 1–3 = mild pain, 4–6 = moderate pain and 7–10 = severe pain.

### 2.6 Foot cooling

Following the CST, participants went to an adjacent room with an ambient temperature of 25.0 (1.2)°C and 47 (9)% relative humidity to determine the vascular responses to foot cooling as described previously (Lunt & Tipton, 2014). In a seated position, with their knees bent at a 90° angle to ensure an even pressure, participants rested their bare feet on an aluminium footplate perfused with water at 35°C to give a footplate temperature of 33.7 (0.5)°C. Following a 10 min stabilisation period, the footplate was cooled at a rate of 1.8 (0.1)°C/min to 15°C or until the participants were no longer able to tolerate the cold. Skin blood flow and temperature of the great toe was recorded continuously from laser Doppler probes (VP1T/7, Moor Instruments) and skin thermistors (EUS‐U, Grant Instruments) placed on the tip and the medial side of the great toe, respectively. For many participants (NFCI 6/15; COLD 8/18; CON 11/18), it was not possible to identify the onset of vasoconstriction by visual inspection of the flux data as described previously (Maley et al., 2014). Therefore, average CVC and great toe skin temperature were taken over the final 5 min of the footplate at 33°C and during cooling at footplate temperatures of 26–25°C, 21–20°C and 16–15°C. Participants’ perception of thermal comfort, sensation and pain of their feet was reported before cooling and with every 5°C fall in plate temperature using the same visual analogue scale as for the CST.

### 2.7 Heart rate variability

R–R interval was recorded during the cutaneous local heating protocol using a heart rate monitor (V800, Polar Electro Oy, Kempele, Finland) when the participants were resting in a seated position and thumb and great toe skin temperatures were clamped to 33°C. Heart rate variability (HRV) was analysed from steady, artefact‐free, 10 min recordings of R–R interval using Kubios HRV Standard program (Ver. 3.1, Kubios Oy, Kuopio, Finland). Frequency domain analysis was conducted and absolute power (ms^2^) for the bands of low frequency (LF; 0.04–0.15 Hz) and high frequency (HF; 0.15–0.4 Hz) were measured and the ratio of LF to HF power was calculated (LF/HF). Relative LF and HF power were also expressed in normalised units (nu) calculated by dividing each power by the total power minus very low frequency power (0–0.04 Hz).

### 2.8 Questionnaires

All participants completed a cold exposure questionnaire (Eglin et al., 2020) that asked for details about their lifetime cold exposure and occupational history (including the use of vibrating tools and chemical exposure). Participants were asked to rate their ability to cope with the cold on a three‐point scale (1 better than average, 2 average and 3 worse than average) for both their whole body and their extremities (hands and feet). All participants also completed the cold intolerance symptom severity (CISS) questionnaire, which was analysed using the scoring system described previously (Carlsson et al., 2010).

Participants in the NFCI group also completed questionnaires relating to any pain they experienced as a consequence of NFCI. These included the Brief Pain Inventory (BPI; Chen et al., 2018), Douleur Neuropathique en 4 (DN4) pain questionnaire (Bouhassira et al., 2005), the short forms of the Pain Catastrophizing Scale (PCS; McWilliams et al., 2015) and Pain Self‐Efficacy Questionnaire (PSEQ; Sullivan et al., 1995). All questionnaires were completed at an ambient temperature of 24°C.

### 2.9 Data analysis

Between‐group analysis was conducted on matched participants only (maximum *n* = 16 for each group). Data collected from the feet or great toe were only analysed on participants matched to individuals with NFCI in their feet (*n* = 13) whereas data collected from the thumb were only analysed in participants matched to individuals with NFCI in their hands (*n* = 11).

The distribution of data was assessed using descriptive methods (skewness, outliers and distribution plots) and inferential statistics (Shapiro–Wilk test). Where normal distribution was violated, non‐parametric analyses were performed. Between‐group analysis was conducted using either a one‐way ANOVA with pairwise comparisons with least significant differences *post hoc* test or a Kruskal–Wallis test with Mann–Whitney *U post hoc* tests. Correlations were conducted using either Pearson's correlation or Spearman's rank correlation.

For the CST, statistical differences were assessed using a mixed model ANOVA (group (NFCI, COLD, CON) × time (pre‐immersion, 5 min, 10 min)) for mean toe, coldest toe and great toe skin temperature and great toe skin blood flow. Toe skin temperature during the foot cooling protocol was analysed using a mixed model ANOVA (group (NFCI, COLD, CON) × footplate temperature (33°C, 25°C, 20°C, 15°C)). Where appropriate, *post hoc* tests were conducted using pairwise comparisons with least significant differences. Cutaneous vascular conductance in the foot cooling protocol and thermal sensation and comfort during both the CST and the foot cooling protocol were analysed across time using Friedman's test and between groups using the Kruskal–Wallis test with Mann–Whitney *U post hoc* tests where appropriate.

Data are presented as means (SD) or medians (interquartile range (IQR)) unless otherwise stated and are available at pure.port@ac.uk. Statistical analysis was performed with SPSS version 26 (IBM Corp., Armonk, NY, USA) and statistical difference and trends towards significance were accepted as *P* < 0.05 and *P* < 0.1, respectively.

## 3 RESULTS

### 3.1 Participants

**FIGURE 1:**
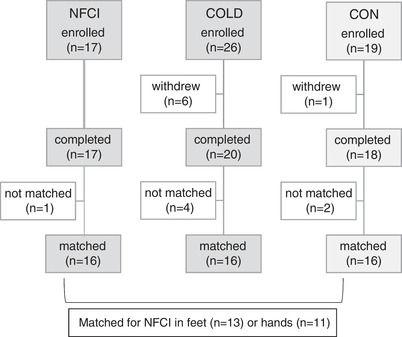
Participant flow through trial. Participant flow through the trial for the three groups. COLD, cold‐exposed controls; CON, non‐cold‐exposed controls; NFCI, individuals with non‐freezing cold injury in their feet and/or hands.

Sixty‐two individuals enrolled in the study, 55 of whom completed the testing between January 2019 and December 2019. A detailed analysis of participants enrolled on to the study is shown in Figure 1. Where participants withdrew from the study after only completing one or two tests, their data were not included in the between‐group analyses whilst those who completed the majority of testing were included. All of the participants who withdrew from the study did so due to unexpected commitments and consequent lack of available time to complete the testing. The three groups – individuals with NFCI (NFCI), cold‐exposed controls (COLD) and non‐cold‐exposed controls (CON) – were closely matched with one White woman and one Caribbean woman in each group. No significant differences were observed between the physical characteristics of each group except for estimated aerobic fitness where CON had a lower Astrand score than NFCI (*P* = 0.025) and COLD (*P* = 0.012; Table 1). All participants had normal blood cell counts, were within the normal range for serum concentrations of cholesterol, triglycerides and HbA1c, and were normotensive.

**TABLE 1:** Participant characteristics.

	NFCI (*n* = 16)	COLD (*n* = 16)	CON (*n* = 16)	*P*
Sex	2 women, 14 men	2 women, 14 men	2 women, 14 men	
Race	8 W, 7 Afr/Car, 1 Mixed	8 W, 7 Afr/Car, 1 Mixed	8 W, 7 Afr/Car, 1 Mixed	
Age (years)	28 (4)	30 (7)	25 (6)	0.072
Height (cm)	176.6 (6.3)	176.1 (7.2)	177.9 (11.4)	0.835
Mass (kg)	75.7 (7.4)	80.5 (11.6)	78.9 (14.0)	0.488
BMI (kg/m^2^)	24.3 (2.1)	25.9 (3.3)	24.7 (1.9)	0.184
Foot volume (cm^3^)	849 (233)	896 (194)	946 (146)	0.471
Estimated ${\dot{V}}_{O_{2}\max}$ (ml/min/kg)	67 (9)*	68 (12)*	58 (11)	**0.022**

Means (SD) are presented for each group. *P*‐values are given for ANOVA; value shown in bold is statistically significant. *Estimated ${\dot{V}}_{O_{2}\max}$ NFCI > CON (*P* = 0.012) and COLD > CON (*P* = 0.025). Afr/Car, African/Caribbean; COLD, cold‐exposed controls; CON, non‐cold‐exposed controls; Mixed, mixed race White/Caribbean; NFCI, individuals with non‐freezing cold injury; W, White.

**FIGURE 2:**
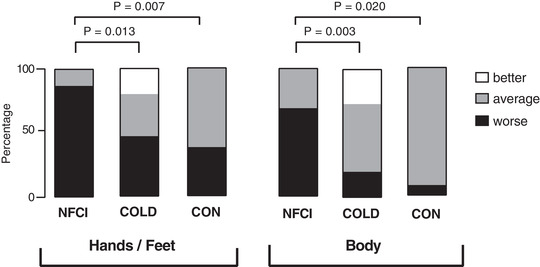
Reported ability to cope with the cold. Reported ability to cope with the cold in the extremities and body in NFCI (individuals with NFCI; *n* = 16), COLD (cold‐exposed controls; *n* = 15), CON (non‐cold‐exposed controls; *n* = 13) from cold exposure questionnaire. Values given are the percentage of the group that thought they coped ‘better than average’ (□), ‘average’ (■) or ‘worse than average’ (■).

Seven of the NFCI (one White, six African/Caribbean), nine of the COLD (one White, one White/Caribbean, seven African/Caribbean) and three of the CON (one White, one White/Caribbean, one African/Caribbean) lived in Africa or the Caribbean as children. The corresponding numbers for living in Europe (mainly UK) as a child were: nine NFCI (seven White, one White/Caribbean, one African/Caribbean); seven COLD (seven White) and nine CON (six White, three African/Caribbean) with the location not being known for four of the CON group. The cold exposure questionnaire indicated that as children, the NFCI group had the least cold exposure and COLD the most with CON being intermediate undertaking outdoor sports such as football (although no statistical analyses were conducted due to the subjective nature of the questionnaire, Table 2). As adults, the NFCI and COLD group reported being exposed to cold, wet conditions for prolonged periods of time during work, whilst only one participant in the CON group reported short (2 h) repeated cold exposure at work. NFCI rated being worse than average at coping with the cold compared with either COLD or CON in both their whole body (*P* = 0.003 and *P* = 0.020) and their extremities (*P* = 0.013 and *P* = 0.007, Figure 2).

**TABLE 2:** Cold exposure during leisure and work.

	NFCI (%) (*n* = 16)	COLD (%) (*n* = 16)	CON (%) (*n* = 12)
<12 year: sports/activities	31	56	42
12–18 year: sports/activities	50	81	58
12–18 year: scouting/adventure sports	31	50	8
Adult: work	94	94	8
Adult: sports/activities	81	94	67
Adult: hobbies	44	69	33

Percentage of participants in each group reporting being exposed to cold, wet conditions during leisure or work activities. No statistical tests were peformed. COLD, cold‐exposed controls; CON, non‐cold‐exposed controls; NFCI, individuals with non‐freezing cold injury.

Five of the NFCI participants had NFCI in their feet only, three in their hands only and eight in their hands and feet. The NFCI group were seen on average 3.9 years (range 2 months to 9.4 years) after their initial injury, which all occurred during military field exercises in freezing conditions (0 to −25°C). Additionally, rain/snow was reported in 11 cases and wind in three. All NFCI reported wearing military‐issued kit at the time of injury, amongst whom five specified cold weather kit, three warm‐weather kit and five combats/issued kit. Following the injurious exposure, 10 NFCI remained in the field for the duration of the task, one was only identified as having a NFCI at the end of the task, one was sent running (however, this made the pain worse), and only two reported being removed immediately once NFCI was identified.

Symptoms of the affected area at the time of injury included numbness (*n* = 12), pain (*n* = 9), paraesthesia (*n* = 3), discomfort (*n* = 3), swelling (*n* = 3) and cold (*n* = 3). This was associated with a change in skin colour in 11 individuals (seven white/pale; three red; two blue/purple). On rewarming, nine NFCI reported pain, six of whom were given pain medication (two participants were taking nortriptyline at the time of testing). Nine NFCI reported having symptoms from their injury when in normal room temperature including numbness (*n* = 6), paraesthesia (*n* = 3), pain (*n* = 2), cold (*n* = 2), aching (*n* = 1), itching (*n* = 1) and swelling (*n* = 1). Seven NFCI reported that their NFCI negatively affected their quality of sleep due to pain and/or cold in the affected area.

Nine (out of 16) NFCI reported experiencing pain in the 24 h prior to the study, three in the hands/fingers and feet/toes, five in the feet/toes only, and one reported pain in the fingers, toes and lower leg. Pain severity and interference as assessed by the Brief Pain Inventory are shown in Table 3. Fourteen NFCI scored 4 or more on the DN4 questionnaire indicating that their pain was likely to be neuropathic in origin. The mean (SD) score for the pain catastrophizing scale was 30.7 (18.5) with seven NFCI having a score of 30 and above representing a clinically relevant level of catastrophizing (Sullivan, 2009). When broken down into the subscales, the mean (SD) score for rumination was 9.9 (6.0), magnification was 6.5 (4.4) and helplessness was 14.3 (8.5). The mean (SD) score for the pain self‐efficacy questionnaire was 33.1 (20.2).

**TABLE 3:** Pain intensity and interference.

Intensity	Pain severity	Activity	Pain interference
Worst	3.80 (2.91)	General activity	3.60 (3.14)
Least	2.33 (3.08)	Mood	3.87 (3.94)
Average	3.60 (2.55)	Walking	1.93 (2.81)
Now	2.33 (2.50)	Work	3.20 (3.59)
** Overall**	**3.02 (2.73)**	Relations	2.87 (3.32)
		Sleep	3.67 (3.81)
		Enjoyment	3.8 (3.87)
		**Overall**	**3.28 (3.46)**

Mean (SD) pain intensity and interference at injury site reported by participants with NFCI using the BPI questionnaire (*n* = 16). Scores are between 0 and 10. For pain severity, they relate to the worst, least and average pain severity experienced in the last 24 h and the pain experienced at the time of completing the questionnaire (now). For pain interference, the scores relate to how much pain has interfered with undertaking general activities, mood, walking ability, work (outside the home and housework), relations with other people, sleep and enjoyment of life in the last 24 h. The overall scores for pain intensity and interference are given in bold.

**FIGURE 3:**
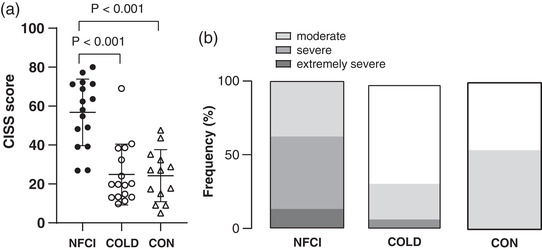
Cold intolerance. (a) Mean (SD) cold intolerance symptoms severity questionnaire (CISS) scores reported by NFCI (individuals with NFCI; *n* = 16), COLD (cold‐exposed controls; *n* = 16) and CON (non‐cold‐exposed controls; *n* = 13). (b) Percentage of the participants who were classified as having mild, moderate, severe or extremely severe cold intolerance for each group (no statistical tests were performed).

Cold intolerance symptom severity (CISS) scores indicated that the NFCI group were more sensitive to the cold than either the COLD or CON (*P* < 0.001; Figure 3a), with 63% of NFCI having severe or extremely severe cold intolerance (Figure 3b). The CISS scores of the CON and COLD groups were similar with neither group having extremely severe cold intolerance and only one participant having severe cold intolerance (Figure 3b).

For the NFCI group, a higher cold intolerance (CISS) was associated with a higher DN4 score (*r*_P_ = 0.709; *P* = 0.002), pain interference (*r*_P_ = 0.812; *P* < 0.001), pain catastrophising (*r*_P_ = 0.636; *P* = 0.011) and a lower pain self‐efficacy (*r*_P_ = −0.747; *P* = 0.001). A low DN4 score was associated with pain interfering less with activities (*r*_P_ = 0.563; *P* = 0.029), lower pain catastrophising (*r*_P_ = 0.571; *P* = 0.026) and with greater pain self‐efficacy (*r*_P_ = −0.650; *P* = 0.009). A positive relationship was observed between pain severity and interference caused by pain (*r*_P_ = 0.729; *P* = 0.002), whereas pain catastrophizing and pain self‐efficacy were inversely related (*r*_P_ = −0.788; *P* < 0.001).

### 3.2 Vascular tests

#### 3.2.1 Deep inspiration

**FIGURE 4:**
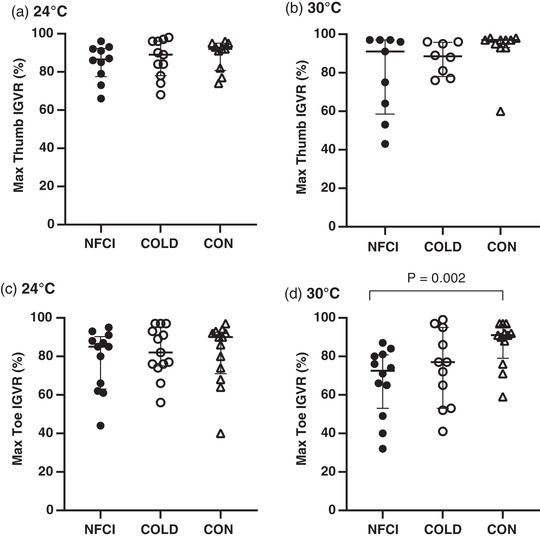
Deep inspiration vascular response. Maximum inspiratory gasp vascular response (IGVR) measured on the thumb (a, b) and great toe (c, d) at an ambient temperature of 24°C (a: NFCI *n* = 10; COLD *n* = 11; CON *n* = 10; c: NFCI *n* = 12; COLD *n* = 13; CON *n* = 13) and 30°C (b: NFCI *n* = 9; COLD *n* = 8; CON *n* = 10; d; NFCI *n* = 12; COLD *n* = 11; CON *n* = 12). Individual data points are shown for each group and the bars represent the median (IQR). The Kruskal–Wallis test indicated no between‐group differences for the thumb at 24° C (*P* = 0.636) or 30°C (*P* = 0.086) or toe at 24°C (*P* = 0.669). COLD, cold‐exposed controls; CON, non‐cold‐exposed controls; NFCI, individuals with non‐freezing cold injury.

Baseline thumb skin blood flow was similar between groups at 24°C (2.06 (0.83) flux/mmHg, *F* = 0.765, *P* = 0.475) and 30°C (2.22 (0.9.) flux/mmHg, *H* = 1.389, *P* = 0.499). Maximum IGVR in the thumb was similar between groups at both ambient temperatures (median (IQR), at 24°C NFCI: 87 (15)%; COLD: 89 (18)%; CON 92 (14)%, *H* = 0.906, *P* = 0.636, Figure 4a; at 30°C NFCI: 91 (39)%; COLD: 88.5 (18)%; CON 97 (4)%, *H* = 4.899, *P* = 0.086; Figure 4b). No differences in hand (*H* = 4.253, *P* = 0.119) or forearm (*F* = 0.718, *P* = 0.490) skin temperatures at 24°C (not measured at 30°C) were seen between groups (Table 4).

**TABLE 4:** Skin temperature during vascular tests.

		Skin temperature (°C)			Skin temperature (°C)	
		Forearm	Hand		Calf	Foot
Deep inspiration						
	*n*	Mean (SD)	Median (IQR)	*n*	Mean (SD)	Mean (SD)
NFCI	10	31.8 (1.1)	31.8 (8.3)	12	29.8 (1.2)**	29.5 (2.1)
COLD	11	32.2 (1.4)	32.7 (2.9)	13	31.2 (1.9)	30.5 (2.3)
CON	7	31.6 (1.3)	31.1 (2.0)	13	32.1 (2.1)	29.6 (1.7)
*P*		0.119	0.490		0.008	0.733
PORH						
	*n*	Mean (SD)	Median (IQR)	*n*	Median (IQR)	Mean (SD)
NFCI	11	31.6 (1.5)	31.7 (7.6)	13	29.3 (2.2)**	28.7 (1.8)
COLD	11	32.7 (0.9)	32.3 (3.2)	13	31.2 (3.7)	30.2 (2.1)
CON	9	32.0 (0.4)	30.7 (1.6)	12	33.2 (2.8)	29.5 (1.8)
*P*		0.054	0.174		0.016	0.155
Cutaneous local heating						
	*n*	Median (IQR)	Median (IQR)	*n*	Median (IQR)	Mean (SD)
NFCI	10	32.0 (2.5)	31.1 (4.8)#	12	29.0 (2.3)*	28.3 (1.7)
COLD	10	32.8 (1.4)*	33.1 (2.9)*	12	31.2 (3.4)*	30.1 (2.2)
CON	8	31.6 (0.9)	29.9 (2.8)	11	33.5 (2.7)	29.1 (1.6)
*P*		0.047	0.027		0.008	0.055
Iontophoresis						
	*n*	Mean (SD)	Mean (SD)	*n*	Mean (SD)	Mean (SD)
NFCI	14	31.6 (1.1)#	29.4 (3.2)	11	28.8 (1.1)	29.5 (2.3)
COLD	14	32.5 (0.8)**	30.8 (2.7)	10	29.2 (1.3)	30.8 (1.8)
CON	14	31.5 (1.0)	30.4 (2.0)	12	29.3 (2.2)	29.0 (2.0)
*P*		0.015	0.356		0.791	0.087

Skin temperature at the forearm, hand, calf and foot during the deep inspiration, post‐occlusive reactive heating (PORH), cutaneous local heating (LH) and iontophoresis protocols. Values are the mean (SD) with *P*‐value from ANOVA or median (IQR) with *P*‐value from Kruskal–Wallis test for the three groups. Significant differences are indicated: *P < 0.05, **P < 0.01 compared to CON; #*P* < 0.05 compared to COLD: deep inspiration: calf – NFCI < CON (*P* = 0.002); PORH: calf – NFCI < CON (*P* = 0.01); LH: forearm – COLD > CON (*P* = 0.013), hand – NFCI < COLD (*P* = 0.031), COLD > CON (*P* = 0.016), calf – NFCI vs. CON (*P* = 0.007), COLD vs. CON (*P* = 0.016); iontophoresis: forearm – NFCI < COLD (*P* = 0.019), COLD > CON (*P* = 0.008). COLD, cold‐exposed controls; CON, non‐cold‐exposed controls; NFCI, individuals with non‐freezing cold injury.

In the great toe, baseline skin blood flow was similar between groups at 24°C (median (IQR) 1.06 (1.05) flux/mmHg, *H* = 4.049, *P* = 0.132) but was greater in COLD compared to CON at 30°C (NFCI: 2.27 (1.4) flux/mmHg; COLD: 2.76 (1.5) flux/mmHg; CON: 1.56 (0.8) flux/mmHg, *P* = 0.016). Maximum great toe IGVR at 24°C was similar between groups (median (IQR), NFCI: 85 (27)%; COLD: 89 (21)%; CON 90 (22)%; *H* = 0.804, *P* = 0.669, Figure 4c). At 30°C, maximum IGVR was significantly lower in NFCI compared to CON (median (IQR): 73 (28)% vs. 91 (17)%; *U* = 20; *P* = 0.003; Figure 4d); however, the IGVR of COLD did not differ from either CON (*P* = 0.169) or NFCI (77 (42)%, *P* = 0.372). Calf skin temperature was lower in NFCI compared to CON (*P* = 0.002) and there was a trend for it being lower than COLD (*P* = 0.058); however, foot skin temperature was similar between groups (*F* = 0.259, *P* = 0.773, Table 4).

### 3.3 Post‐occlusive reactive hyperaemia

**FIGURE 5:**
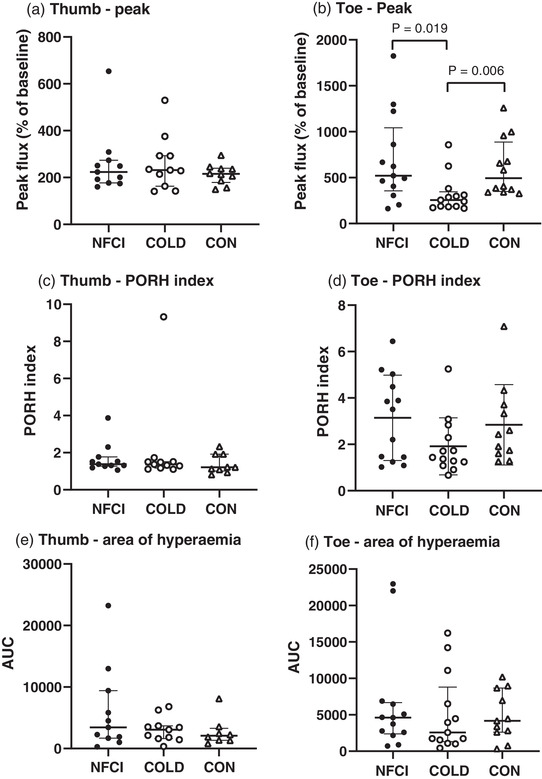
Post‐occlusive reactive hyperaemia. Skin blood flow responses following occlusion of the thumb (a, c, e) and great toe (b, d, f). Individual values and median (IQR) are shown for the peak flux as a percentage of the baseline (a, b), PORH index (c, d) and the hyperaemic response (e, f). NFCI (individuals with NFCI ●, thumb *n* = 11; toe *n* = 13), COLD (cold‐exposed controls ○, thumb *n* = 11; toe *n* = 13) and CON (non‐cold‐exposed controls △, thumb *n* = 9; toe *n* = 12). No between‐group differences were seen in thumb for peak (*P* = 0.765), PORH index (*P* = 0.458) or area of hyperaemia (*P* = 0.520) or in toe for PORH index (*P* = 0.130) or area of hyperaemia (*P* = 0.739) using the Kruskal–Wallis test.

Peak flux in the great toe following occlusion was significantly lower in COLD (median (IQR) 257 (161)%) compared to either CON (494 (537)%; *U* = 20, *P* = 0.002) or NFCI (520 (687)%; *U* = 36, *P* = 0.013); however, PORH index (*H* = 4.083, *P* = 0.130) and area of hyperaemia (*H* = 0.606, *P* = 0.739) were similar between groups (Figure 5). Compared to CON, calf skin temperature was 3.9°C cooler in NFCI (*U* = 30.5, *P* = 0.01) and 2.0°C cooler in COLD though this did not reach significance (*U* = 43, *P* = 0.057; Table 5). Foot skin temperature was similar between groups (*F* = 1.970, *P* = 0.155; Table 5).

**TABLE 5:** Cutaneous local heating.

	Thumb				Great toe			
	NFCI (*n* = 10)	COLD (*n* = 10)	CON (*n* = 8)	*P*	NFCI (*n* = 12)	COLD (*n* = 11)	CON (*n* = 11)	*P*
Peak (%)	249 (232)	176 (108)	217 (183)	0.458	657 (539)	294 (476)	679 (193)	0.131
Nadir (%)	137 (135)	100 (32)	106 (50)	0.150	308 (505)	220 (198)	377 (246)	0.601
42°C plateau (%)	150 (130)	113 (42)	110 (68)	0.208	434 (422)	236 (272)	374 (287)	0.612
44°C plateau (%)	169 (158)	128 (61)	152 (104)	0.317	499 (616)	251 (520)	406 (454)	0.455
AUC ratio	1.95 (1.50)	1.20 (0.60)	1.40 (0.6)	0.187	4.62 (5.20)	2.75 (2.30)	4.16 (2.00)	0.409

Median (IQR) of thumb and great toe skin blood flow parameters measured during cutaneous local heating in each of the groups. Peak, nadir and plateaux values are presented as a percentage of baseline CVC and the ratio between AUC at baseline CVC and during the first 20 min of hyperaemia is also given. *P*‐values are from the Kruskal–Wallis test. AUC, area under the curve; COLD, cold‐exposed controls; CON, non‐cold‐exposed controls; NFCI, individuals with non‐freezing cold injury.

In the thumb, peak flux (*H* = 0.536, *P* = 0.765), PORH index (*H* = 1.560, *P* = 0.458) and area of hyperaemia (*H* = 1.308, *P* = 0.520) were similar between groups (Figure 5). Forearm and hand skin temperature were also similar (*F* = 3.245, *P* = 0.052 and *H* = 3.493, *P* = 0.174, respectively; Table 5) and MAP did not differ (*F* = 0.368, *P* = 0.695) between groups during the protocol (NFCI: 87 (8) mmHg; COLD: 85 (7) mmHg; CON: 86 (6) mmHg).

### 3.4 Cutaneous local heating

In the great toe, baseline CVC was lower in CON compared to COLD (median (IQR) 0.50 (0.23) flux/mmHg vs. 0.95 (1.54) flux/mmHg; *U* = 31, *P* = 0.011); however, NFCI (0.61 (1.05) flux/mmHg) was not significantly different from either COLD (*P* = 0.103) or CON (*P* = 0.386). Baseline thumb CVC values were similar between groups (mean (SD): NFCI = 1.23 (0.87) flux/mmHg; COLD = 1.98 (0.91) flux/mmHg; CON = 1.31 (0.88) flux/mmHg, *F* = 2.135, *P* = 0.139). No differences were observed between groups for any of the parameters measured during cutaneous local heating in either the toe or thumb when expressed as percentage or ratio of baseline CVC (Table 5).

Foot skin temperature was not significantly different between groups (*F* = 3.18, *P* = 0.055, Table 4). Calf skin temperature was highest in the CON group (*H* = 9.747, *P* = 0.008), being 4.5°C warmer than NFCI (*U* = 22, *P* = 0.007) and 2.3°C warmer than COLD (*U* = 27, *P* = 0.016, Table 4). In contrast, forearm and hand skin temperatures were significantly warmer in COLD compared to CON (*U* = 12, *P* = 0.013 and *U* = 13, *P* = 0.016, respectively, Table 4). Hand skin temperature was also cooler in NFCI compared to COLD (*U* = 21.5, *P* = 0.031, Table 4).

### 3.5 Iontophoresis

**FIGURE 6:**
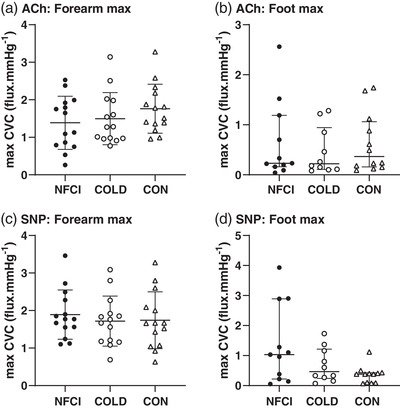
Response to ACh and SNP. Maximum cutaneous vascular conductance (CVC) to iontophoresis of acetylcholine (ACh, a, b) and sodium nitroprusside (SNP, c, d) on the forearm (a, c) and foot (b, d) for each group (NFCI: ● individuals with NFCI; COLD: ○ cold‐exposed controls; CON: △ non cold‐exposed controls). Individual values with mean (SD) following a cumulative current of 600 μA (*n* = 14 for each group) are shown for the forearm and median (IQR) following a cumulative current of 100 μA (NFCI *n* = 11, COLD *n* = 9, CON *n* = 12) for the foot. No between‐group differences were seen with ANOVAs in the forearm for ACh (*P* = 0.749) or SNP (*P* = 0.196) or with the Kruskal–Wallis test in the foot for ACh (*P* = 0.343) or SNP (*P* = 0.777).

No differences in maximum vasodilatory response to ACh or SNP in the forearm (ACh: *F* = 1.100, *P* = 0.343; SNP: *F* = 0.254, *P* = 0.777) or foot (ACh: *H* = 0.579, *P* = 0.749; SNP: *H* = 3.256, *P* = 0.196) were observed between groups (Figure 6). AUC for ACh and SNP also did not differ between groups in the forearm (mean (SD) for ACh: 6.80 (3.84) arbitrary units (au); *F* = 0.627, *P* = 0.539; SNP: 6.90 (3.54) au; *F* = 0.356, *P* = 0.702) or foot (ACh: 1.30 (1.50) au, *H* = 0.321, *P* = 0.852; SNP: 1.29 (1.54) au; *H* = 3.003, *P* = 0.223).

Hand, calf and foot skin temperatures were similar between groups (hand: *F* = 1.060, *P* = 0.356; calf: *F* = 0.236, *P* = 0.791; foot: *F* = 2.657, *P* = 0.087); however, forearm skin temperature was warmer in COLD compared to NFCI (*P* = 0.019) or CON (*P* = 0.008; Table 4).

### 3.6 Cold sensitivity test

**FIGURE 7:**
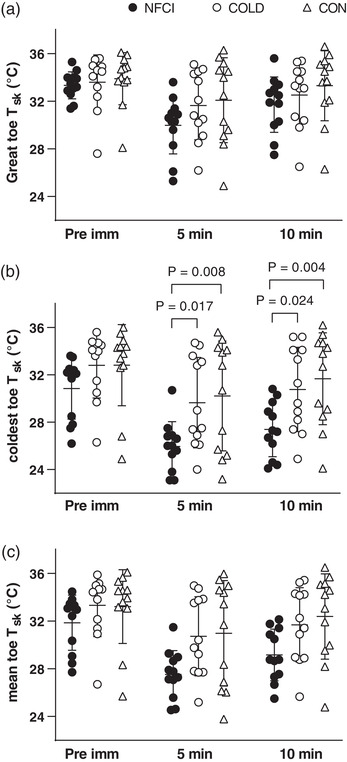
Skin temperature during CST. Skin temperature of the great toe (a), coldest toe (b) and mean of all toes (c) during the cold sensitivity test for each group (NFCI: ● individuals with NFCI, *n* = 12; COLD: ○ cold‐exposed controls, *n* = 11; CON: △ non cold‐exposed controls, *n* = 12). Individual data and mean (SD) are shown for before immersion (Pre imm), 5 min of rewarming (5 min) and 10 min of rewarming (10 min). ANOVA showed no between‐group differences for great toe (*P* = 0.361) or mean toe (*P* = 0.059) skin temperature; pre‐immersion the coldest toe skin temperature did not differ between groups (*P* = 0.722).

Following foot immersion, toe skin temperatures (great toe, coldest toe and mean toe) decreased and were significantly lower at 5 min of rewarm compared to pre‐immersion (*P* < 0.001). Although skin temperature increased significantly at 10 min of rewarm, these were still below the pre‐immersion temperature (*P* < 0.001, Figure 7). A significant difference between groups was only observed in the coldest toe skin temperature (*F* = 4.408, *P* = 0.02), which was lower in NFCI compared to both COLD and CON at 5 min (*P* = 0.017 and *P* = 0.008) and 10 min (*P* = 0.024 and *P* = 0.004; Figure 7b). No differences were observed between the skin temperatures of COLD and CON (*P* = 0.722).

Cutaneous vascular conductance measured at the great toe was significantly greater pre‐immersion (mean (SD): 2.9 (1.6) flux/mmHg) compared to that at 5 and 10 min post‐immersion/of rewarming (1.9 (1.4) and 2.2 (1.6) flux/mmHg, *F* = 31.9, *P* < 0.001), but did not differ between groups at any time point (*F* = 0.355, *P* = 0.704).

**FIGURE 8:**
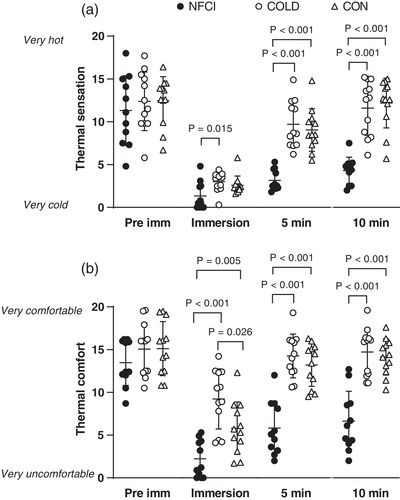
Thermal sensation and comfort during CST. Thermal sensation (a) and thermal comfort (b) for the foot during the CST for each group (NFCI: ● individuals with NFCI, *n* = 12; COLD: ○ cold‐exposed controls, *n* = 11; CON: △ non‐cold‐exposed controls, *n* = 12). Individual data and mean (SD) are shown for before immersion (Pre imm), immersion, 5 min of rewarming (5 min) and 10 min of rewarming (10 min). Kruskal–Wallis test showed no between‐group differences pre‐immersion for thermal sensation (*P* = 0.776) or thermal comfort (*P* = 0.318).

Prior to immersion, thermal sensation and comfort of the foot was similar between groups (*H* = 2.292, *P* = 0.318, Figure 8). On immersion, the NFCI group reported their foot felt colder than COLD (*P* = 0.015; Figure 8a) but not CON and less comfortable than both groups (COLD: *P* < 0.001 and CON: *P* = 0.017, Figure 8b). During the rewarming, NFCI participants reported that their foot felt colder and more uncomfortable compared to both COLD and CON (*P* < 0.001; Figure 8). Although thermal sensation was similar (*P* = 0.903), CON reported their foot felt more uncomfortable during immersion compared to COLD (*P* = 0.026). During rewarming, no differences were observed in the reported foot thermal sensation or comfort between COLD and CON (*P* = 0.355 and *P* = 0.544, respectively, Figure 8).

Subjective pain in the foot was reported more commonly in the NFCI group than either the COLD or CON group during the CST. In CON, no pain was reported before the immersion or during the rewarm period; however, five of the 13 participants reported pain during foot immersion (two pain score 1/10, two 3/10 and one 4/10). There were no reports of pain before immersion in the COLD group, with one participant reporting a pain score of 3/10 during immersion and another reporting pain at both 5 and 10 min of rewarming (5/10 and 1/10, respectively). In the NFCI group, four participants reported no pain during the CST, and three participants reported pain during immersion (2 = 3/10; 6/10) and at 5 min of rewarm (all 1/10) with no pain being reported by 10 min rewarming. Two participants reported pain during the immersion and throughout the rewarming period (immersion: 6/10, 3/10; 5 min of rewarming: 4/10, 3/10; 10 min of rewarming: 1/10, 2/10). The remaining three participants reported pain in their foot throughout the CST (pre: 1/10, 2/10, 3/10; immersion: 3/10, 4/10, 4/10; 5 min rewarming: 2/10, 3/10, 3/10; 10 min rewarming: 2/10, 3/10, 3/10).

In the NFCI group, total pain reported during the CST (the sum of the four time points) showed a significant positive moderate correlation with their BPI interference score (*r*_s_ = 0.598, *P* = 0.040).

### 3.7 Foot cooling

Great toe skin blood flow and temperature were similar between groups (*H* = 2.316, *P* = 0.314 and *F* = 1.610, *P* = 0.217, respectively) when the footplate was maintained at 33°C and during the subsequent cooling period (Table 6). As expected, both CVC and skin temperature significantly decreased over time (χ^2^ = 40, *P* < 0.001 and *F* = 142, *P* < 0.001, respectively).

**TABLE 6:** Foot cooling.

		Foot plate temperature			
		33°C	25°C	20°C	15°C
Foot skin temperature (°C)	NFCI	29.9 (1.3)	28.6 (1.1)	26.7 (1.0)	24.7 (0.8)
	COLD	30.9 (1.9)	29.9 (1.4)	28.4 (1.2)	25.8 (1.5)
	CON	29.3 (2.7)	28.6 (2.6)	27.5 (2.6)	25.3 (2.9)
	ANOVA	temperature × group *P* = 0.217			
Foot skin blood flow (flux/mmHg)	NFCI	0.89 (0.74)	0.46 (0.34)	0.38 (0.45)	0.32 (0.27)
	COLD	1.45 (1.24)	1.13 (1.02)	1.04 (1.22)	0.58 (0.72)
	CON	0.87 (0.91)	0.74 (0.77)	0.64 (0.77)	0.63 (0.83)
	K‐Wallis	*P* = 0.314	*P* = 0.531	*P* = 0.186	*P* = 0.449
Thermal sensation (0–20)	NFCI	9.5 (3.8)*#	3.2 (2.7)	1.9 (2.2)**#	0.2 (0.4)***##
	COLD	13.6 (2.7)	5.9 (4.4)	5.2 (4.4)	3.6 (4.9)
	CON	12.9 (3.2)	4.7 (1.7)	3.5 (1.4)	1.4 (0.8)
	K‐Wallis	*P* = 0.035	*P* = 0.081	*P* = 0.021	*P* < 0.001
Thermal comfort (0–20)	NFCI	11.3 (4.0)	5.7 (4.1)#	4.1 (4.2)*#	1.3 (1.8)**#
	COLD	13.5 (3.7)	8.3 (3.4)	7.9 (2.8)	5.8 (3.5)
	CON	12.9 (3.9)	10.1 (2.7)	7.9 (3.0)	3.8 (2.5)
	K‐Wallis	*P* = 0.235	*P* = 0.026	*P* = 0.023	*P* = 0.005

Mean (SD) foot skin temperature, cutaneous vascular conductance, thermal comfort and sensation reported during the foot cooling protocol for NFCI (individuals with NFCI, *n* = 12), COLD (cold‐exposed controls, *n* = 12) and CON (non‐cold‐exposed controls, *n* = 12). Three participants in the NFCI group stopped the test before the footplate temperature reached 15°C and therefore *n* = 9 at this time point. *P*‐values are given for ANOVA and Kruskal–Wallis (K‐Wallis) tests. *Post hoc* Mann–Whitney *U*‐test for thermal sensation at 33°C: NFCI < COLD (*P* = 0.023), NFCI < CON (*P* = 0.039); 20°C: NFCI < COLD (*P* = 0.009), NFCI < CON (*P* = 0.049); and 15°C: NFCI < COLD (*P* = 0.001), NFCI < CON (*P* = 0.002). *Post hoc* Mann–Whitney *U*‐test for thermal comfort at 25°C: NFCI < CON (*P* = 0.013); 20°C NFCI < COLD (*P* = 0.015), NFCI < CON (*P* = 0.021); and 15°C NFCI < COLD (*P* = 0.004), NFCI < CON (*P* = 0.017). *P < 0.05, **P < 0.01, ***P < 0.001 compared to COLD; #*P* < 0.05, ##*P* < 0.01 compared to CON.

Participants reported the foot feeling increasingly cold (χ^2^ = 83, *P* < 0.001, Table 6) and less comfortable (χ^2^ = 60, *P* < 0.001, Table 6) as the footplate cooled. At footplate temperatures of 33°C (baseline), 20°C and 15°C but not 25°C, NFCI reported having colder feet than either COLD or CON (33°C: *U* = 32.5, *P* = 0.023 and *U* = 36, *P* = 0.039; 20°C: *U* = 27, *P* = 0.009 and *U* = 38, *P* = 0.049; 15°C: *U* = 5, *P* < 0.001 and *U* = 11.5, *P* = 0.002, respectively). No differences in thermal sensation were observed between COLD and CON at any footplate temperature (*P* = 0.128 to *P* = 0.977).

Thermal comfort at baseline did not differ between groups (*H* = 2.893, *P* = 0.235) but at a footplate temperature of 25°C NFCI reported greater discomfort than CON (*U* = 29, *P* = 0.013). NFCI also reported greater thermal discomfort of the foot compared to COLD and CON at a footplate temperature of 20°C (*U* = 30, *P* = 0.015; *U* = 32, *P* = 0.021) and 15°C (*U* = 13.5, *P* = 0.004; *U* = 20.5, *P* = 0.0217; Table 6). No differences in thermal comfort were observed between COLD and CON at any footplate temperature (*P* = 0.198 to *P* = 0.887).

Subjective pain in the foot was reported more commonly in the NFCI group than either the COLD or CON group during the foot cooling protocol with three participants from the NFCI group withdrawing before the footplate temperature reached 15°C. At a footplate temperature of 25°C none of the CON group reported pain, compared to two participants from COLD (1/10 and 2/10) and eight from the NFCI group (1–4/10). At a footplate temperature of 20°C there was one report of pain in the CON group (1/10), two from the COLD group (2/10) and nine from the NFCI group (1–4/10). In the NFCI group, total pain reported during the foot cooling protocol was correlated with pain reported during the CST (*r*_s_ = 0.636, *P* = 0.026) and pain severity as assessed by the BPI questionnaire (*r*_s_ = 0.641, *P* = 0.025) but no other assessments of pain or cold intolerance.

### 3.8 Heart rate variability

LF power (1906 (1022) ms^2^, *H* = 5.21, *P* = 0.074; 70.0 (16.9) nu, *F* = 1.000, *P* = 0.378), HF power (1194 (1861) ms^2^, *H* = 4.400, *P* = 0.108; 29.9 (16.9) nu, *F* = 1.000, *P* = 0.379) and LF/HF ratio (3.69 (3.27), *H* = 0.780, *P* = 0.676) were not different between groups.

## 4 DISCUSSION

This is the first study to undertake a detailed examination of vascular function in individuals with NFCI and matched control groups who had either experienced similar exposure to cold (COLD) or minimal cold exposure (CON). It was hypothesised that the NFCI would show impaired vascular function compared to the control groups. NFCI showed a decreased sensitivity to sympathetic vasoconstrictor activation in the toe (IGVR at 30°C) and greater cold sensitivity (CST) compared to COLD and CON. Given that none of the other tests of vascular function (PORH, cutaneous local heating, vasodilatory response to ACh and SNP or foot cooling) indicated endothelial dysfunction, the hypothesis can only be partially accepted. However, despite the small differences in skin temperature, NFCI perceived their feet were much colder and more uncomfortable during the CST and foot cooling protocol and were more likely to report pain than either control group.

The three groups were closely matched for physical characteristics including sex, race, age, height, mass, BMI and foot volume (Table 1). Although CON had a lower estimated ${\dot{V}}_{O_{2}\max}$ compared to NFCI and COLD, there was considerable overlap between groups (range: NFCI = 51–77 ml/min/kg; COLD = 48–83 ml/min/kg; CON = 47–70 ml/min/kg). Since none of the participants in the CON group were classified sedentary, it is not thought that this would impact on the responses to the vascular tests (Simmons et al., 2011). The NFCI and COLD group had similar cold exposure at work as both groups were employed by the British Army in contrast to the CON group who had minimal cold exposure at work (Table 2). More of the COLD group reported undertaking sports and activities in cold/wet conditions as children and adults compared to either the NFCI or CON group. The reason for this is not known: in theory, early frequent exposure might have been protective. Alternatively, self‐selection (those best able to tolerate exposure continued to do it) may have occurred.

The NFCI group were seen on average 3.3 years (2 months to 9.4 years) after their initial injury and were all in the chronic phase of their injury, which was caused by exposure to freezing and often wet conditions. The symptoms presented by the NFCI group were similar to those reported in recent studies of NFCI (Vale et al., 2017; Kuht et al., 2018) with pain and sensitivity to cold being the main symptoms. The mean pain intensity was 3.0 and that for pain interference was 3.3 from the BPI questionnaire (Table 3), which was lower than the 6.0 and 7.2 reported by Vale *et al.* (2017). Similarly, whilst the NFCI patients in the study by Vale *et al.* (2017) all had a DN4 score of ≥4, which is indicative of neuropathic pain, 14 out of the 16 NFCI had a score of ≥4 in our study. This would indicate that our NFCI group had a less severe form of NFCI, which is perhaps unsurprising since our patients were recruited from regional NFCI clinics rather than the specialist neuropathic clinics of Vale et al., (2017). As such, they may be more common and representative of NFCI patients in general.

Seven of the NFCI had a total PCS score of 30, indicating a clinically relevant level of catastrophizing (Sullivan, 2009). A high PCS score has been found to predict the development of chronic pain (Picavet et al., 2002). Pain self‐efficacy refers to beliefs that one can enjoy life and engage in activities despite perceiving pain (McWilliams et al., 2015). The average score reported by NFCI was 33, which was similar to that reported by individuals with chronic pain with neuropathic characteristics (Torrance et al., 2013). Low pain self‐efficacy is associated with a feeling that the pain is uncontrollable and unmanageable during daily life (Linton & Shaw, 2011). Scores below 30 are associated with long‐term disability and depression (Arnstein et al., 1999) and it is of concern that seven NFCI scored below this level. This indicates the degree to which NFCI can negatively impact an individual's quality of life.

The CISS and cold exposure questionnaires revealed that NFCI were more cold intolerant/less able to cope with the cold in their extremities and body than either the CON or COLD group, who were similar (Figure 3). The mean CISS score for COLD and CON was 27, which is higher than the normative value of 13 reported for individuals with no medical history of upper extremity injury or Raynaud's phenomenon by Ruijs et al. (2006). However, this may not be an appropriate comparison as the participants in this study (Ruijs et al., 2006) were from northern Sweden, were older with a greater BMI and were predominantly (62%) women. All of these factors influence cold sensitivity as assessed by the CISS questionnaire (Ruijs et al., 2006). The fact that a greater proportion of the COLD group seemed to undertake activities exposing themselves to cold/wet environments suggests that there was an element of self‐selection. In addition, individuals within the NFCI group who reported cold intolerance had greater pain catastrophizing and lower pain self‐efficacy. Severity and symptoms of pain assessed through either the DN4 or BPI questionnaires correlated with the interference the pain caused to everyday activities. The correlations suggest that those individuals with NFCI who perceived that they were intolerant to cold experienced more pain. This in turn, had a greater impact on their daily activities and was associated with increased pain catastrophization and reduced ability to cope with the pain.

Deep inspiration causes an increase in sympathetic outflow resulting in peripheral vasoconstriction (Mayrovitz & Groseclose, 2002), which is attenuated in conditions such as diabetes that are associated with vascular dysfunction (Aso et al., 1997). In the current study, deep inspiration resulted in a reduction in cutaneous blood flow in the thumb and great toe (Figure 5); however, no significant difference between groups was observed when at an ambient temperature of 24°C (Figures 4a and c), supporting earlier work in patients with sequelae from local cold injuries (probably frostbite; Arvesen et al., 1994). Deep inspiration was also conducted at a warmer ambient temperature after gentle exercise to remove central vasoconstrictor tone (Eglin et al., 2013), which may reduce the IGVR. A reduced toe IGVR was observed in NFCI compared to CON (Figure 4d) indicating a reduced sensitivity to sympathetic activation.

Heart rate variability (HRV) is a measure of neurocardiac function reflecting heart–brain interactions and the dynamic nature of the autonomic nervous system. Over the relatively short time periods that HRV was measured during rest in the current study, power in the HF band represents respiratory sinus arrhythmia due to efferent parasympathetic activity and that in the LF band to baroreflex activity (Shaffer & Ginsberg, 2017). The NFCI group did not differ from either of the control groups for any of the HRV indices. Therefore, these results (deep inspiration and HRV) indicate that the increased cold intolerance reported by NFCI was probably not related to a general increase in sympathetic tone or sensitivity to sympathetic stimulation.

Great toe peak blood flow following occlusion was reduced in COLD compared to the other two groups (Figure 5b), suggesting that cold exposure in the absence of cold injury may attenuate vasodilator prostaglandin activity (Binggeli et al., 2003). However, this was not supported by other measures of reactive hyperaemia (PORH index or area of hyperaemia, Figure 5) or seen in the thumb.

The vasodilatory response to cutaneous heating is mediated by a variety of different mechanisms, and can thus be useful in characterising endothelial dysfunction. The initial peak in cutaneous blood flow is mediated by a sensory nerve axon reflex (Hodges et al., 2015) involving adenosine receptors (Fieger & Wong, 2010), TRPV‐1 channels (Fieger & Wong, 2010) and endothelium‐derived hyperpolarising factor (EDHF) (Brunt & Minson, 2012), whilst the sustained vasodilatory plateau is mediated by nitric oxide (NO) (Minson et al., 2002) and EDHF (Brunt & Minson, 2012). In the current study, the vasodilatory response to local heating was not reduced in either the NFCI or COLD group compared to CON (Table 5) suggesting that these vasodilatory pathways are not impaired. This is in contrast to individuals with secondary Raynaud's phenomenon (Boignard et al., 2005) or those who are older (Minson et al., 2002) or smokers (Avery et al., 2009) where a reduced vasodilatory response is observed. In addition, African American individuals have an attenuated response to local heating compared to White individuals due to reduced NO bioavailability (Kim et al., 2018). It is well known that African/Caribbean individuals are at higher risk of NFCI (Kuht et al., 2018), and this is reflected in the high proportion of this race in the NFCI group (53%) in relation to the proportion enrolled in the British Army (7%). However, since each of our groups was matched for race and age, these factors were controlled for. Although there were differences between groups in the skin temperatures measured during the PORH, cutaneous local heating and forearm iontophoresis tests (Table 4), this is not thought to be a large confounding factor as skin temperature at the site of the laser Doppler probe was clamped with the local heater.

No difference in endothelium‐dependent or ‐independent vasodilatation as assessed by iontophoresis of ACh and SNP, respectively, was observed between groups at either the forearm or foot (Figure 6). Previous studies have shown ACh‐mediated vasodilatation to be attenuated in African individuals when compared to White individuals (Maley et al., 2015) and in patients with Raynaud's (Gardner‐Medwin et al., 2001), but not in individuals who are cold sensitive (Eglin et al., 2020). This indicates that NFCI is not associated with cutaneous vascular dysfunction either systemically (forearm) or close to the site of injury (foot). However, there are some limitations to the method of iontophoresis which involves transdermal delivery of the drug into the epidermis through electrical repulsion. Although a known current can be applied to a known concentration of the drug, the delivery of the drug is not known and is affected by cutaneous blood flow (Roustit et al., 2014). Drug delivery is also affected by skin resistance which is higher in black skin (Pienaar et al., 2014). This means that not all of the participants could receive the maximum current of 200 μA. The vasodilatory response to ACh is endothelium‐dependent, involving a variety of mediators including NO (Kellogg et al., 1999), prostaglandin (Maley et al., 2017) and EDHF release (Brunt et al., 2015). Whilst the NO‐mediated vasodilatory response to ACh is reduced in black individuals, this is compensated by an upregulation of EDHF (Ozkor et al., 2014), and therefore, although the response to ACh was similar in NFCI, COLD and CON, the mechanisms producing that vasodilatation could be different. In addition, the high level of aerobic fitness in the NFCI group may have had a protective effect on vasodilatory function (Kvernmo et al., 1998) and therefore, in less fit individuals with NFCI, vascular dysfunction may be more apparent.

The cold sensitivity test has been used previously in cold‐injury clinics for the diagnosis of NFCI (House et al., 2015), with a cold hand or foot skin temperature at an ambient temperature of 30°C and a slow rate of rewarming following a short cold exposure (2 min immersion in 15°C water) indicating cold sensitivity. However, a wide range of responses are observed in an apparently ‘normal’ uninjured population (Tipton et al., 2020; Haman *et al.*, 2022), with individuals undertaking recreational activities such as windsurfing and open water swimming being cold sensitive in the absence of a NFCI (Eglin, 2011; Hope et al., 2014; Eglin et al., 2020). In control participants, skin rewarming following the cold challenge is mostly due to the return of skin blood flow. NFCI patients with severe cold sensitivity rewarm at the same rate as a control individual when the blood flow to their foot is occluded (Davey et al., 2013). This indicates that cold sensitivity is a result of reduced peripheral skin blood flow, which may be due to a reduction in NO availability as glyceryl trinitrate was found to increase the rate of rewarming in cold sensitive, but not control, individuals (Hope et al., 2014).

In the current study, the skin temperature of the coldest toe was significantly lower in NFCI during the rewarming period compared to COLD and CON (Figure 7b), indicating a greater degree of cold sensitivity. Accompanying the slower rate of toe rewarming, the NFCI group perceived their foot as colder, more uncomfortable and painful than either the CON or COLD groups (Figure 8). During the footplate cooling test, toe skin temperature and blood flow were similar between groups (Table 6); despite this, the NFCI group again perceived their feet as colder, more uncomfortable and painful than either the CON or COLD groups (Table 6). This indicates that the perception of skin temperature and associated pain may be altered with NFCI. This is investigated further in the following paper, where the thermal detection and pain thresholds were examined in the three groups.

One of the potential limitations of this study was that the CON group were tested when the outside ambient temperature was warmer compared to the NFCI or COLD group. Seasonal differences in the response to cold have been reported, with the metabolic response to cool air exposure being greater in winter compared to summer although no differences in body temperature were observed (van Ooijen et al., 2004). Lower finger skin temperature has been reported in winter compared to summer in some studies (Gardner‐Medwin et al., 2001) but the converse has also been found (Makinen et al., 2004). We are confident that the outside ambient temperature is unlikely to have had a large effect on our results. Firstly, whilst we did see some significant differences in skin temperature during the vascular tests (Table 4), generally foot and hand skin temperature of COLD was greater than either NFCI or CON indicating outside temperature was not a factor. Secondly, the room temperature in which the tests were conducted was controlled and the same for all groups. Thirdly, the participants undertook a period of acclimatisation of at least 30 min prior to testing and in the CST, which is thought to be particularly sensitive to varying levels of vasoconstrictor tone, light exercise was conducted prior to the acclimation period. Finally, during the measurement of CVC in the deep inspiration, PORH, cutaneous local heating and iontophoresis protocols, the skin temperature at the site of measurement was clamped with an integrated heater. For logistic reasons, it was not possible to conduct the tests at the same time of day and therefore circadian effects could have masked between‐group differences.

In this study, we examined vascular function in individuals with NFCI compared with two matched control groups with either similar (COLD) or minimal (CON) cold exposure. Individuals with NFCI in their feet appeared to have greater cold sensitivity compared to either CON or COLD and this was associated with an augmented sensation of cold, thermal discomfort and pain. It is unlikely that this was due to increased sympathetic activity since the response to deep inspiration was reduced in NFCI and no differences in HRV were observed between groups. Vascular function as assessed by reactive hyperaemia, local heating and transdermal delivery of ACh and SNP does not appear to be impaired in individuals with NFCI in either their hands or feet. However, there is considerable redundancy in the mechanisms which result in vasodilatation, meaning that whilst the net vasodilatory response has not been changed with NFCI, the mechanism through which it has been produced may be altered. This is examined in the third paper in this series where pertinent blood biomarkers in each group are investigated. As NFCI is thought to be a vaso‐neuropathy it is important that the neural consequences of NFCI are also investigated and this is addressed in the next paper.

## AUTHOR CONTRIBUTIONS

Data were collected during experiments performed in laboratories at the University of Portsmouth and Catterick Garrison. Clare Eglin, Heather Massey, Sarah Hollis, Matthew Maley, Hugh Montgomery and Michael Tipton were involved in the concept and design of the work. Clare Eglin, Jennifer Wright, Heather Massey, Matthew Maley, Hugh Montgomery and Michael Tipton were involved the acquisition, analysis or interpretation of the data. Clare Eglin produced the first draft of the manuscript, all authors revised it critically and approved the final version of the manuscript and agree to be accountable for all aspects of the work in ensuring that questions related to the accuracy or integrity of any part of the work are appropriately investigated and resolved. All persons designated as authors qualify for authorship, and all those who qualify for authorship are listed.

## CONFLICT OF INTEREST

None of the authors have any conflicts of interest.

## Supporting information

Statistical Summary Document

## Data Availability

The data that support the findings of this study are available at https://researchportal.port.ac.uk/en/datasets/dataset-for-the-peripheral-vascular-responses-in-non-freezing-col (https://doi.org/10.17029/c4cf7f6b-223f-4c3c-8864-b1fefdc2e5b8).
